# Resistance of seagrass habitats to ocean acidification via altered interactions in a tri-trophic chain

**DOI:** 10.1038/s41598-020-61753-1

**Published:** 2020-03-20

**Authors:** Begoña Martínez-Crego, Salvatrice Vizzini, Gianmaria Califano, Alexia Massa-Gallucci, Cristina Andolina, Maria Cristina Gambi, Rui Santos

**Affiliations:** 1 University of Algarve (UAlg-CCMAR), Campus de Gambelas, 8005-139 Faro, Portugal; 2 University of Palermo, Department of Earth and Marine Sciences, Via Archirafi 18, 90123 Palermo, Italy; 3 CoNISMa, National Inter-University Consortium for Marine Sciences, Piazzale Flaminio 9, 00196 Roma, Italy; 4 Friederich-Schiller-University Jena (FSU), Lessingstr. 8, D-07743 Jena, Germany; 5 Stazione Zoologica Anton Dohrn, Department of Integrative Marine Ecology, Villa Dohrn. Benthic Ecology Center (Ischia), Villa Comunale, 80121 Naples, Italy; 6 AquaBioTech Group, Central Complex, Naggar Street Targa Gap, Mosta, MST 1761 Malta

**Keywords:** Marine biology, Environmental impact, Climate-change ecology, Ecosystem ecology, Stable isotope analysis

## Abstract

Despite the wide knowledge about prevalent effects of ocean acidification on single species, the consequences on species interactions that may promote or prevent habitat shifts are still poorly understood. Using natural CO_2_ vents, we investigated changes in a key tri-trophic chain embedded within all its natural complexity in seagrass systems. We found that seagrass habitats remain stable at vents despite the changes in their tri-trophic components. Under high *p*CO_2_, the feeding of a key herbivore (sea urchin) on a less palatable seagrass and its associated epiphytes decreased, whereas the feeding on higher-palatable green algae increased. We also observed a doubled density of a predatory wrasse under acidified conditions. Bottom-up CO_2_ effects interact with top-down control by predators to maintain the abundance of sea urchin populations under ambient and acidified conditions. The weakened urchin herbivory on a seagrass that was subjected to an intense fish herbivory at vents compensates the overall herbivory pressure on the habitat-forming seagrass. Overall plasticity of the studied system components may contribute to prevent habitat loss and to stabilize the system under acidified conditions. Thus, preserving the network of species interactions in seagrass ecosystems may help to minimize the impacts of ocean acidification in near-future oceans.

## Introduction

Ecological systems are organized in more or less complex networks of species interactions; being the strength of such interactions critical on how the system is structured and respond to environmental change^1–3^. Ocean acidification (OA) due to increasing human emissions of carbon dioxide (CO_2_) into the atmosphere is causing unequal positive or negative impacts on different species^4,5^, thus changing the strength of their interactions with largely unpredictable effects on ecosystems^6^.

Of particular concern are OA-driven changes on foundation (habitat-forming) species, since they may modify habitat structural complexity and the environmental context in which other species interact. Most studies on kelp forests and coral reefs showed major shifts to simplified habitats dominated by turf-algae^7,8^. A boosted primary productivity of certain non-calcifying species, which are able to benefit from both the increase in inorganic carbon availability for photosynthesis and the reduction in the abundance of dominant calcareous producers, is consistently reported^9–11^. However, reports are not uniform regarding the control exerted by consumers on the bloomed productivity. Some experiments^8^ and observations at CO_2_ vents^12^ indicate that under acidified conditions the enhanced resource effect of high *p*CO_2_ on turf algae, combined with a weakened top-down control by herbivores (reduced abundance and/or feeding), drive the reorganization of kelp forests towards turf-dominated systems. Other mesocosm experiments suggest that an amplified herbivory control on the boosted productivity may alternatively stabilize the system^13,14^. In both cases, herbivory plays a central role in promoting the system resistance or change since it acts as connector of bottom-up (resource-driven) and top-down processes (consumer-driven).

In iconic seagrass systems, shifts to algal-dominated habitats are often associated to a reduced grazing by small invertebrate herbivores on epiphytes and opportunistic algae, whilst shifts to unvegetated habitats are associated to extremely high herbivory events by sea turtles or sea urchins (review by Maxwell *et al*.^15^). Such shifts are largely due to eutrophication and/or the removal of predators that control herbivore populations by overfishing, either directly or indirectly via reduced control on small predators that feed on algae-removing mesograzers. We know comparatively less about changes in the strength of species interactions that may critically influence the persistence of seagrass ecosystems under ocean acidification.

The Mediterranean endemic seagrass *Posidonia oceanica* forms complex systems with well-defined main trophic links. Two macroherbivores alone, the sea urchin *Paracentrotus lividus* and the sparid fish *Sarpa salpa* (commonly known as salema), may remove 50% of the annual seagrass productivity in shallow meadows^16^. In this study, we investigate mechanisms behind the change or stability of *Posidonia* habitats under ocean acidification. Particularly, we examined the interactions’ strength under present (off-vent) and near-future OA conditions (CO_2_ vents) of a tri-trophic food chain embedded within all its natural complexity. We studied multiple basal resources, one of the two main seagrass herbivores (sea urchin), and a territorial labrid fish that is known to predate on such herbivore^17,18^ (*Symphodus tinca*, commonly known as peacock wrasse). Both consumers have restricted benthic home ranges, which ensures long-term exposure to high *p*CO_2_ levels at the vent sites for them and their resources. Particularly, we investigated the strength of consumers’ feeding by quantifying CNP stoichiometry, diet composition, trophic niche and position (stable isotope analysis (SIA)-based and diet-based), as well as the availability and palatability of resources to herbivores. We also examined the strength of the predation pressure mediated by both, direct (predator abundance) and indirect interactions (habitat structure influencing refuge provision and fish herbivory as modifier of such structure). Herbivore abundance, resulting from the propagation of both, resource- and predator-driven effects, was also quantified.

The most recent experiments on OA research (e.g. refs. ^8,19^) incorporate a few species interactions and a longer exposure to CO_2_ enrichment than the conventional experiments, which are typically restricted to short-term exposure of a single species or trophic level. Still, laboratory or mesocosm experiments cannot reflect the full complexity of the interactions’ network, nor the full compensatory and adaptive potential of species gained across generations. On the contrary, the use of natural CO_2_ vents to replicate future OA effects on ecosystems overcomes such experimental constraints, in spite of some limitations such as the relatively small spatial scales and open nature of vent systems.

## Results

### Herbivore feeding: food abundance and quality

**Figure 1:**
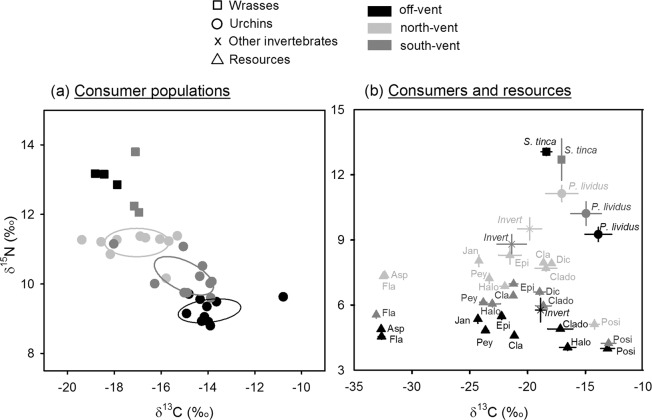
Trophic structure of consumer populations based on isotopic signatures at off-vent and vent sites. (**a**) δ^13^C vs. δ^15^N of consumers (single replicates) with solid lines enclosing the corrected standard ellipse areas (=isotopic niche widths) of urchin populations, where the diversity of resources exploited (δ^13^C range), the trophic niche redundancy (packing) and length (δ^15^N range), as well as the position of the niche centroid (which changes indicates trophic plasticity), are visualized for off-vent to south-vent and north-vent replicates. (**b**) δ^13^C vs. δ^15^N of basal resources and consumers (‰, mean ± standard deviation). Resources are indicated as follows: Asp, *Asparagopsis armata*; Clado, *Cladophora prolifera*; Cla, *Cladophora* spp.; Dic, *Dictyota* spp.; Epi, Epiphytes Fla, *Flabellia petiolata*; Halo, *Halopteris scoparia*; Jan, *Jania rubens*; Pey, *Peyssonnelia* spp.; Posi: *Posidonia oceanica*. Data on other invertebrate (*Invert*) obtained from Ricevuto *et al*.^43^ in the same sites and season were used exclusively as basal resources for wrasses in the SIA-based calculations. Values of the metrics of trophic structure and statistical comparison among sites are shown in Supplementary Table S4 (sea urchins) and S5 (wrasses). Statistical differences in isotopic signatures are shown in Supplementary Tables S6 (resources) and S7 (consumers).

**Figure 2:**
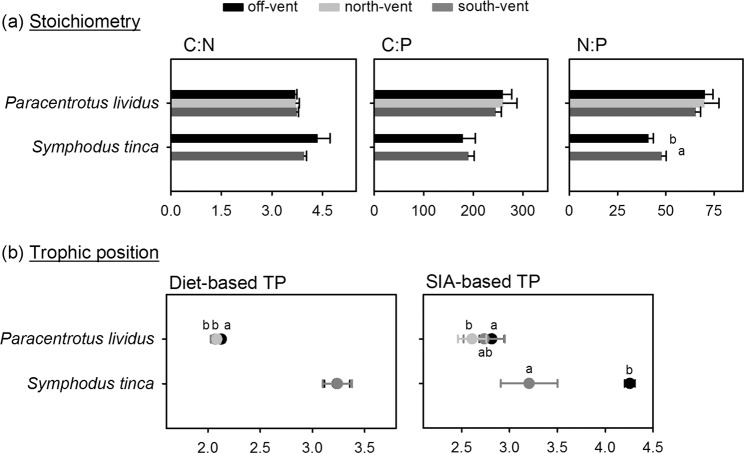
C:N:P stoichiometry (**a**) and trophic position (**b**) of consumers at off-vent and vent sites (mean ± standard deviation). Trophic positions (TP) were obtained using methods based on diet composition (left graph) and stable isotopes (right graph). Different letters above bars or symbols denote significant differences among sites based on post hoc comparisons from one-way PERMANOVAs. Elemental contents are shown in Supplementary Fig. S1 and detailed statistical results in Supplementary Tables S8 (stoichiometry) and S9 (TP).

We detected no differences between sites in the niche width or in the number of carbon resources exploited by sea urchin populations, which indeed showed similar Standard Ellipse Areas and bootstrapped Layman metrics (Fig. 1a). In contrast, the isotopic niche centroid of sea urchins differed among sites (PERMANOVA Pseudo-F = 26, p = 0.001), occupying the most δ^15^N depleted and δ^13^C enriched position at off-vent followed by south-vent and with north-vent occupying the opposite position (Fig. 1, PERMANOVA p = 0.0001 for resource x site interaction in both, δ^15^N and δ^13^C). No difference in sea urchin stoichiometry was found between sites (Fig. 2a; PERMANOVA non-significant p-values for CNP contents and ratios).

**Figure 3:**
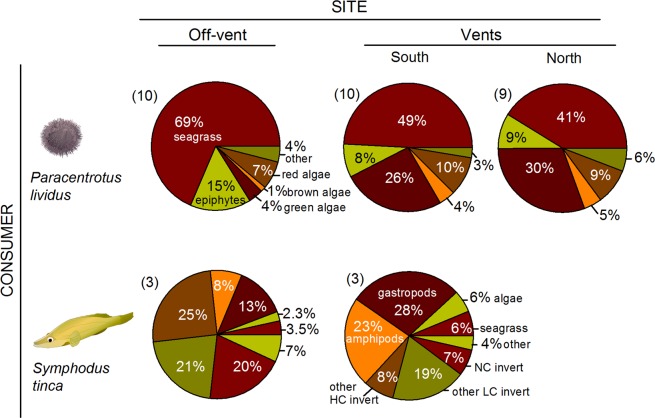
Mean diet composition of consumers at off-vent and vent sites. For illustrative purposes food items were grouped as detailed in SIMPER results shown in Supplementary Tables S10 (sea urchins) and S11 (wrasses). Abbreviations are as follows: Non-calcified benthic invertebrates (NC invert); Less calcified benthic invertebrates (LC invert); Heavily calcified benthic invertebrates (HC invert); Unidentified material (Other). Colour legend of food items is shown in one pie chart for each consumer species. The number of replicates is indicated in the parentheses. Data of *P. lividus* diet obtained from Nogueira *et al*.^24^. Statistical differences in diet composition, which are indicative of consumer trophic plasticity, are shown in Supplementary Table S12. Consumer images are courtesy of the Integration and Application Network, University of Maryland Center for Environmental Science (ian.umces.edu/symbols/).

**Figure 4:**
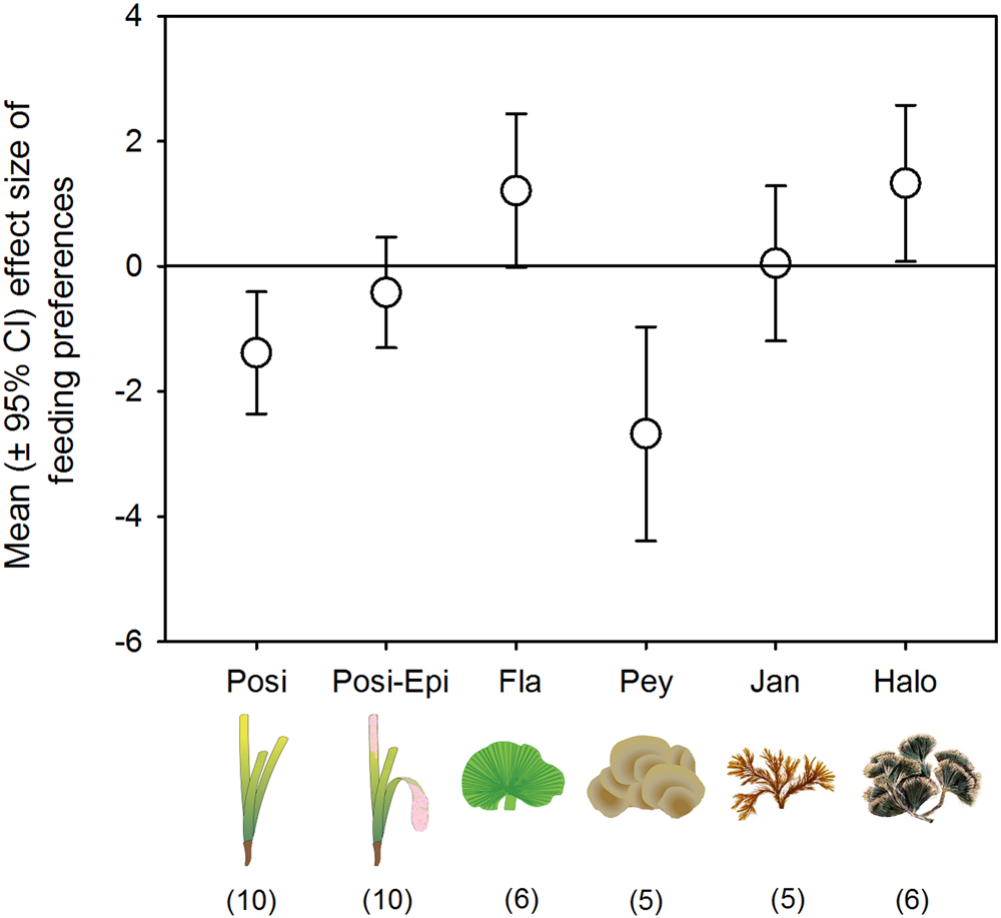
Feeding preference of sea urchins toward resources from off-vent *versus* vent sites (mean ± 95% CI). Effects are significantly different from zero if CIs do not overlap with zero. Negative values indicate preference for off-vent material, while positive values illustrate significant preference for vent material. The number of assays used in each estimate is indicated in the parentheses. Resources are indicated as follows: Posi: *Posidonia oceanica*; Posi-Epi, *Posidonia* with epiphytes, Fla, *Flabellia petiolata*; Pey, *Peyssonnelia* spp.; Jan, *Jania* rubens; Halo, *Halopteris scoparia*. Detailed consumptions are shown in Supplementary Fig. S2. Images were courtesy of the Integration and Application Network (ian.umces.edu/symbols/) or generated by BMC using SigmaPlot v11.1.

**Figure 5:**
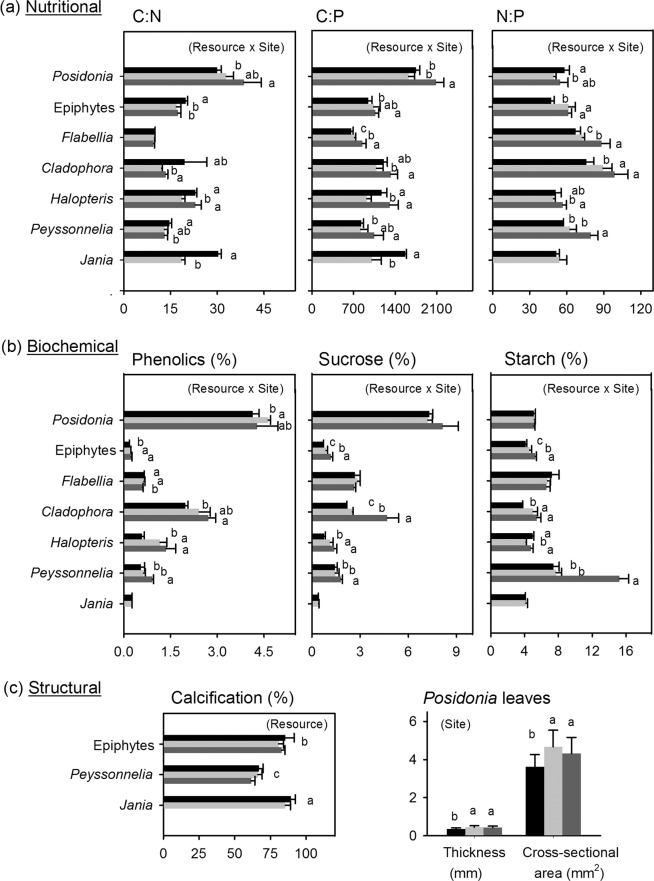
Nutritional, chemical and structural quality of the most abundant resources available to herbivores at off-vent (black) and vents (dark grey: south-vent, light grey: north-vent). Data are mean ± standard deviation (n = 4, except n = 5 for *Posidonia* and *Peyssonnelia* phenolics). Significant effects from PERMANOVAs are shown within the parenthesis. Different letters next to bars denote significant differences among sites for a given resource or for each *Posidonia* structural trait. Statistics are detailed in Supplementary Tables S13 and S14, and elemental contents in Supplementary Fig. S1.

Diet-based trophic position of sea urchins was lower at vents than at off-vent (Fig. 2b; PERMANOVA Pseudo-F = 8.8, p = 0.001) due to a lower ingestion of seagrass and calcareous epiphytes (50–57% at vents *vs*. 84% at off-vent) at both vents, where the green algae *Flabellia* and *Cladophora* were more consumed (ca. 25% at vents *versus* 3% at off-vent; see Fig. 3, diet composition PERMANOVA Pseudo-F = 6.9, p = 0.0001; pairwise comparisons: off-vent ≠ north- ~ south-vent). Using the SIA-based method, a lower trophic position of sea urchins was found only at north-vent than at off-vent (PERMANOVA Pseudo-F = 3.8, p = 0.03). This reflects a lower detection ability of the SIA method likely related to the appropriate choice of baseline references, which is particularly tricky for herbivores since basal resources have a high interspecific isotopic variability^20^. These changes in diet agreed with the resource palatability that we observed in feeding assays, as sea urchins preferred off-vent *Posidonia* and vent *Flabellia* (Fig. 4). The lower preference for vent *Posidonia* was likely related to its higher leaf thickness and cross-sectional area (Fig. 5c), since the seagrass nutritional and chemical traits that we measured did not consistently vary between vent and off-vent sites (Fig. 5a,b). The preference for off-vent *Posidonia* disappeared when epiphytes were present in seagrass leaves. Seagrass epiphytes showed higher nutritional quality at both vents than off-vent (higher N:P and C, N and P content, and lower C:N; higher sucrose and starch as follows south-> north-> off-vent), but also higher phenolics. Among algal resources with an amplified contribution to urchin diet at vents, *Cladophora*’s nutritional quality was higher at both vents than off-vent (higher N, N:P, sucrose and starch; Fig. 5). Urchin preference on other algae was not related to the food quality traits that we measured, i.e. the sea urchins preference for vent *Flabellia* with lower P content and higher C:P and N:P; for vent *Halopteris* with higher sucrose, N and P contents, but also higher C and phenolics; for off-vent *Peyssonnelia* with lower N content; and no preference for vent *Jania* with higher N and P contents and lower C:N and C:P. Calcification degree significantly varied between calcareous resources (*Jania* > Epiphytes > *Peyssonnelia*; PERMANOVA p = 0.0001 for resource), with a tendency (PERMANOVA p = 0.05 for site) to lowered calcification at vents (Fig. 5c).

The diversity of food resources available to herbivores, as well as the abundance of the common algae *Flabellia*, *Halopteris* and *Peyssonnelia*, was similar at vents and off-vent (Table 1). *Posidonia* was more abundant at both vents, where it had less epiphytes, particularly at south-vent. *Cladophora* was scarce at off-vent and slightly more abundant at both vents. *Jania* was very scarce at the south-vent (found in sea urchin guts but not in the meadow), while at north-vent and off-vent it was also found as epiphyte of *Flabellia* and *Halopteris*. *Asparagopsis* was particularly abundant in shallow areas at the north-vent and absent at south-vent, whereas only the tetrasporophyte (“Falkenbergia-phase”) of this alga was found off-vent. Other scarce algae, which also appeared in sea urchin’s diet, were *Cladophora* spp. and *Dictyota* spp., with the later absent off-vent. The green algae *Caulerpa cylindracea* (all sites), *Anadyomene stellata* (off-vent, south-vent), and *Bryopsis* spp (off-vent, north-vent) were very scarce in the meadows and absent in consumers’ diet.

**Table 1:** Availability of food resources to herbivores at off-vent and vent sites.

Resource	Off-vent	North-vent	South-vent
*Posidonia oceanica*	++	+++	+++
Seagrass epiphytes	+++	++	+
*Flabellia petiolata*	+++	+++	+++
*Halopteris scoparia*	+++	+++	+++
*Peyssonnelia* spp.	+++	+++	+++
*Jania rubens*	+++	+++	−^(a)^
*Cladophora prolifera*	+	++	++
*Asparagopsis armata*	+^(b)^	++	−
*Cladophora* spp.	+	+	+
*Dictyota* spp.	−	+	+
*Anadyomene stellata*^(c)^	+	−	+
*Bryopsis* spp^(c)^	+	+	−
*Caulerpa cylindracea*^(c)^	+	+	+
DIVERSITY OF FOOD RESOURCES	12	12	10(11)*

The abundance of algal resources and epiphytes was estimated within four 15 m^2^ areas per site as follows: +++ = very abundant; ++ = less abundant; + = very scarce; − = absent. Abundance of seagrass where obtained from the literature^20,57,62^. Notes are as follows: ^(a)^*Jania* was absent in south-vent samples (not collected for analyses), but was present in urchin guts; ^(b)^Only the tetrasporic phase of *Asparagopsis* (previously named “Falkenbergia”) was found at the off-vent meadow, the gametophytic phase was collected from an adjacent rocky substract; ^(c)^*Caulerpa cylindracea* (all sites), *Anadyomene stellata* (off-vent, south-vent) and *Bryopsis* spp. (off-vent, north-vent) were very scarce and absent in consumer diets, then they were not further processed. *Resource diversity considering items in sea urchin guts is shown between brackets.

### Predator feeding

We observed a relatively higher degree of omnivory (NR) and similitude in trophic habits of wrasses (SDNND), as well as lower diversity of carbon resources exploited (CR), at south-vent than off-vent; but these were single values not tested for significance (Fig. 1). The position of the niche centroid of wrasse populations did not significantly differ between vents and off-vent (Fig. 1a; PERMANOVA Pseudo-F = 3.6, p = 0.09), despite the depleted δ^13^C values found at the off-vent (Fig. 1b; PERMANOVA Pseudo-F = 22, p = 0.01). Wrasse stoichiometry did not vary between sites, with the exception of a higher N:P ratio at the south-vent than off-vent (Fig. 2a; PERMANOVA Pseudo-F = 13, p = 0.02). The SIA-based trophic position of wrasses was higher at off-vent than at south-vent (PERMANOVA Pseudo-F = 37, p = 0.004), while the diet-based trophic position did not significantly vary between sites (Fig. 2b; PERMANOVA Pseudo-F = 0.001, p = 0.98). Site-dissimilarity in wrasse diet was also relatively high (58%; mainly due to a higher ingestion of gastropods and amphipods at south-vent instead of foraminifera, isopods, sipunculid worms, and bivalves at off-vent, see Fig. 3), but differences were not significant due to a high variability among the low number of replicates (diet composition PERMANOVA Pseudo-F = 2.0, p = 0.15).

### Herbivore and predator abundance

**Figure 6:**
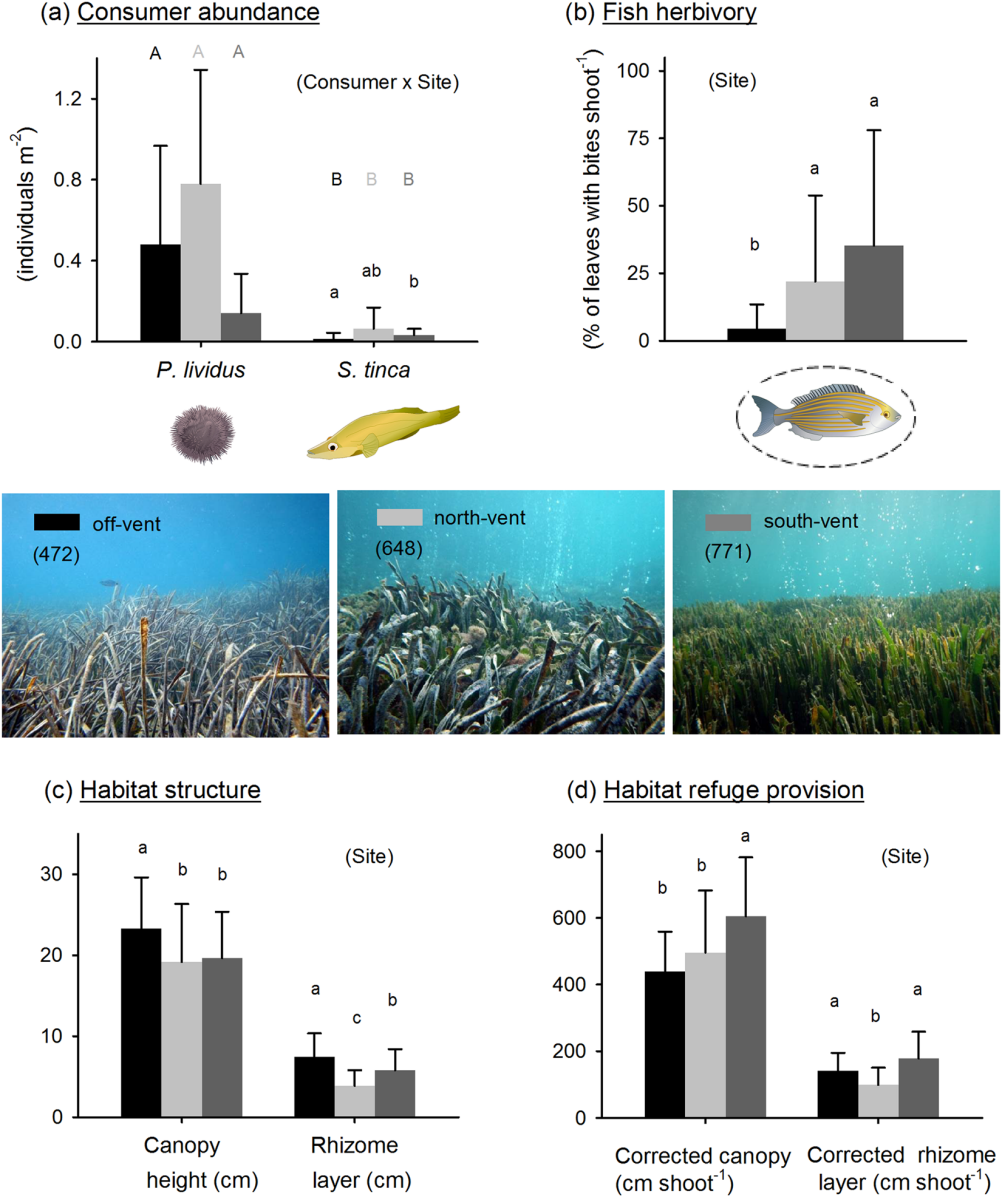
Consumer abundances (**a**), fish herbivory (**b**), habitat structure (**c**) and refuge provision (**d**) at off-vent and vent sites. Data are mean ± standard deviation. PERMANOVA significant effects are shown within the parenthesis. Lowercase letters denote significant differences among sites. Capital letters denote significant differences between urchin and wrasse abundances within each site following the colour legend shown in the pictures. Statistics are detailed in Supplementary Tables S15 and S16. Pictures show the contrasting habitat structural complexity at vents (low canopy height due to an intense fish herbivory) and off-vent (regular canopy height). Mean seagrass density (shoots m^−2^), used to estimate indicators of refuge provision, is indicated in the parentheses within each site picture. Consumer images are courtesy of the Integration and Application Network (ian.umces.edu/symbols/).

We found no differences between vents and off-vent in sea urchin abundance (Fig. 6a). Regardless of the site, sea urchins were more abundant than wrasses, while predator abundance was significantly higher at the south-vent with a more than doubled density than off-vent (mean ± SD: 0.03 ± 0.03 and 0.01 ± 0.03 individuals m^−2^, respectively; PERMANOVA consumer x site interaction Pseudo-F = 2.7, p = 0.03). Albeit not statistically significant due to a high variability in wrasse distribution, the same trend with even a higher mean (0.06 ± 0.11 individuals m^−2^) was observed at the north-vent (Fig. 6a).

### Fish herbivory, habitat structure and refuge provision

Seagrass shoots at both vent sites showed clear signs of intense fish herbivory, with leaves in each shoot much more eaten by salemas than at off-vent (35 ± 43% bitten leaves at south-vent and 22 ± 32% at north-vent *versus* 4 ± 9% at off-vent; see Fig. 6b, PERMANOVA Pseudo-F = 4.1, p = 0.01). This intense fish herbivory resulted in a significantly lower canopy height at both vents (Fig. 6c; PERMANOVA Pseudo-F = 3.1, p = 0.0498), where the rhizome layer was as well lower (being also lower at north- than at south-vent; PERMANOVA Pseudo-F = 13, p = 0.0001). Whilst habitat complexity in terms of canopy height and rhizome layer was lower at vents than off-vent (Fig. 6c), such differences were not consistent in terms of refuge provision (Fig. 6d). Indicators of habitat refuge provision corrected by the number of shoots showed a relatively lower corrected canopy height off-vent and north-vent than south-vent (PERMANOVA Pseudo-F = 6.6, p = 0.003), with the later also showing the highest corrected rhizome layer followed by off-vent (slightly not significantly different than south-vent; pairwise comparison p = 0.057) and with north-vent having the lowest value (PERMANOVA Pseudo-F = 10, p = 0.0004).

## Discussion

Our study revealed that a key seagrass herbivore adjusts its feeding according to CO_2_/OA-mediated changes in the palatability of primary producers, thus maintaining its stoichiometry and trophic niche packing. Consumption by sea urchins was reduced on a less palatable seagrass and its epiphytes, while increased on higher-palatable green algae. Such urchin trophic plasticity reduced herbivory pressure on a seagrass that was subjected to a 5 to 8-fold higher fish herbivory under acidified conditions. This supports the hypothesis that the trophic plasticity of key herbivores may behave as a compensatory mechanism that helps to counteract habitat change and to promote system persistence under OA, as previously suggested for kelp forests^14^.

The constancy in consumer stoichiometry from off-vent to vents agrees with the Ecological Stoichiometry theory, by which elemental ratios of consumers remain constant despite the response of primary producers to changes in the availability of environmental resources^21,22^. This consistency was also observed in the trophic structure of sea urchin populations, as reflected by the similar niche width, number of basal resources exploited, trophic length, and packing at vents and off-vent. Sea urchin trophic plasticity was reflected by a shifted niche position at vents, where sea urchins reduced their feeding on *Posidonia* leaves and epiphytes that naturally dominate their diet (e.g. our results, ref. ^23^). Seagrass with epiphytes from off-vent and vents were equally palatable to sea urchins, while epiphyte removal shifted urchin preference towards off-vent seagrass. The intense fish herbivory at vents mainly removes the apical older parts of *Posidonia* leaves heavily colonized by epiphytes^24^, likely driving the lower seagrass ingestion via either, the loss of higher-quality epiphytes or the lowered palatability of a structurally resistant (thicker) seagrass at vents. Previous studies support this urchin feeding choices on *Posidonia* as mediated by both, epiphyte presence^25,26^ and seagrass structural defences^25,27^. Also, an experimental study conducted on the seagrass *Cymodocea nodosa* showed that *P. lividus* was deterred by CO_2_-enriched plants that were offered without epiphytes, likely due to changes in unidentified structural traits of the seagrass^28^. To overcome the CO_2_-mediated changes in the quality of their food resources at vents, sea urchins increased the ingestion of more palatable *Cladophora* and *Flabellia*. Whilst *Flabellia* was a common green alga elsewhere, *Cladophor*a was less abundant at vents and scarce at off-vent. Thus, the amplified trophic role of these green algae was due to the reduced palatability of the main food source (namely, the seagrass), rather than a bloomed availability as often reported for turf-algae^14^. Concurrently, the feeding on algae instead on seagrass epiphytes at vents drives a lower urchin trophic position, with less degree of omnivory since epiphytes include a mix of algal and animal material (hydrozoans, bryozoans, polychaetes, ascidians, among others; see refs. ^29,30^).

We observed doubled predatory fish densities under acidified conditions, likely due to bottom-up benefits of high *p*CO_2_ on preys, or some other variables that we did not specifically measure. Our finding agrees with other studies in CO_2_ vents reporting an increased biomass of common site-attached carnivorous blennies or triplefins, which was related to a lower abundance of their predators^31^ or to the CO_2_-resource effect^32^. Wrasses at vents exploited a lower diversity of resources and occupied a lower SIA-based trophic position (higher degree of omnivory due to an amplified role of basal resources) than off-vent, although the first observation was constrained by a low replication. Contrastingly to SIA-based results, we found no difference between vent and off-vent in the diet-based trophic positions of wrasses obtained using the diet-based method. Such discrepancies are likely related to intrinsic methodological differences, since SIA indicates assimilated food over weeks-months and gut content analyses a snapshot of recently ingested diet biased towards less digestible resources that remain longer in the stomach^33^. This seems particularly relevant for omnivores/predators that feed on food items entailing a high degree of variation in size and digestibility. We found that the diet of this predatory fish was dominated by amphipods and gastropods at vents (51% of the diet *versus* 21% at off-vent), which have been reported to double their number at these vent sites^34^. Garrard *et al*.^34^ also found a reduced number of bivalve and isopod species under acidified conditions, which may explain their lower contribution and the lower diversity of food resources in wrasse diet at vents.

**Figure 7:**
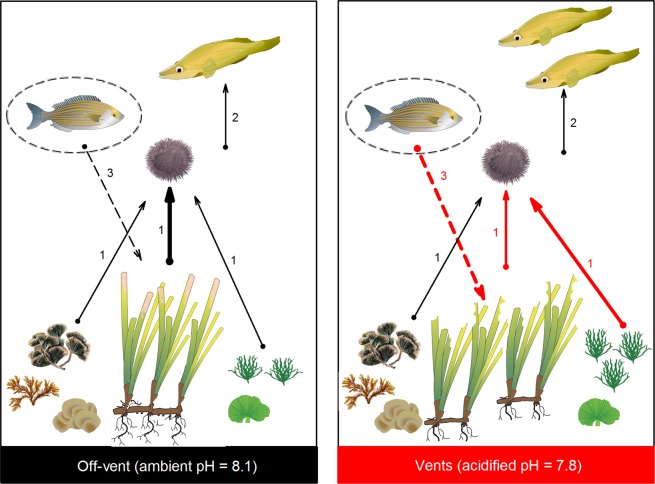
Overview of the tri-trophic seagrass system under ambient (off-vent) and acidified (vent) conditions. The acidified conditions (vents) are characterized by an altered palatability of primary resources (with similar diversity and more abundant *Cladophora* and seagrass but with less epiphytes), with a subsequent adjusted herbivory by sea urchins that is reduced on a thicker seagrass and its epiphytes and amplified on the higher-palatable green algae *Cladophora* and *Flabellia*. Predatory fish populations double their abundances under acidified conditions. Lower habitat structural complexity at vents (i.e. lower seagrass canopy height due to an intense fish herbivory, and lower rhizome layer) is counteracted by a higher shoot density in terms of refuge provision. No change in sea urchin abundance is detected. Arrows represent direct trophic interactions (solid lines numbered as 1: herbivory, 2: predation) and indirect interactions (dashed line numbered as 3: fish herbivory as modifier of habitat structure, namely the seagrass canopy height), being the width of the arrow proportional to the strength of the interaction. Altered interactions (numbers and arrows) in acidified conditions are highlighted in red. Images were courtesy of the Integration and Application Network (ian.umces.edu/symbols/) or generated by BMC using SigmaPlot v11.1.

The abundance of sea urchins did not significantly differ at vents and off-vent, which agrees with previous observations in tropical and temperate vents under near-future OA scenarios^35^. The CO_2_-resource effect on herbivore abundance or a lacking cascade-effect from the increased predator density at vents are likely behind such observation, as summarized in Fig. 7. Indeed, the studied predatory fish fed on several preys other than sea urchins, thereby diluting the predation pressure in multiple directions. We also highlighted that a higher seagrass density at the south-vent compensates the lower seagrass canopy and rhizome layer that we found, thus helping to maintain the refuge provision to sea urchins. Population-level responses to OA may result, as well, from the combined effect of several other factors that we did not specifically measure, such as the evolutionary adaptation of urchin populations at vents via natural selection of resistant phenotypes^36^. Differential species sensitivities to stressor effects of lowered pH on calcification, acid-based regulations, or sensorial and behavioural functioning (e.g. altered prey escape response or predator detection of preys and attack speed) may also contribute to alter the size of herbivore populations, both directly (causing individuals’ mortality) and indirectly (modifying the strength of the top-down control by predators; see reviews ^37,38^).

Our observations at vents showed that OA-driven changes in plant or meadow traits may result in a severe fish herbivory that reduces the height of the seagrass canopy. Studies conducted at these vent sites in different years suggest that the intense fish herbivory that we observed is consistent over the years and significantly different from several control sites^39,40^. Furthermore, similar increased salema herbivory on seagrass has also been reported in other shallow Mediterranean vent systems^41^. This suggests that shallow meadows could be subjected to a substantial fish herbivory in near-future oceans. In the studied Ischia vent, fish herbivory is not causing shoot mortality or habitat shift. Indeed, it seems not to counteract the benefits of high *p*CO_2_, as reflected by the relatively higher seagrass shoot density at vents compared to various control sites reported by other studies^34,39,40^. We evidenced that the trophic plasticity of sea urchin populations at vents (reduced feeding on the habitat-forming seagrass) may act as a stabilizing mechanism of a system altered by an intense fish herbivory, thus helping to reinforce the system resistance to OA. This highlights that conservation efforts aimed at safeguarding the natural complexity of species interactions may help to minimize OA impacts in near-future oceans.

## Methods

### Study sites

The study was conducted during October 2013 in *Posidonia* meadows at 2.5–3 m depth in CO_2_-vent and off-vent sites along the northeast coast of the Ischia Island (Italy, NW Mediterranean Sea). Vent sites were at the north and south sides of the islet Castello Aragonese, which are 150 m apart one from each other and separated by a land bridge connecting the Castello islet to Ischia (40 °43′55.5/51.2″N, 13 °57′50.5/47.6″E). The off-vent site was Lacco-Ameno (40 °45′26.0″ N, 13 °53′4.5″ E), located away from any vent influence (6 km from vents) and representative of other control sites reported elsewhere regarding resource availability, stable isotopes of resources and consumers, or urchin abundances^40,42,43^. The pH level represented current conditions at the off-vent site and projections for 2100 in pH reduction at the vent sites (−0.30 to −0.32 pH units under the RCP8.5 scenario^44^; Supplementary Table S1). The gradient of volcanic CO_2_ bubbling at vent sites extended from 0.5 m to 3 m depth. Vent emissions are well characterized and release almost exclusively CO_2_ (with no toxic hydrogen sulphide) at ambient temperature^45,46^. Seawater pH and *p*CO2 measures at the vent sites are consistent over years and differ significantly from control sites^40,42,46^. The pH range of short-term fluctuations is similar to ranges naturally found in other marine habitats such as coral reefs or upwelling areas^47^.

### Sample collection

The abundance of seagrass epiphytes and algal resources was estimated within four zones of ca. 15 m^2^ per site. Within each zone, algal samples were collected in amounts proportional to their abundance by pooling together in a bag samples equivalent to four quadrats of 20 cm ×20 cm, as well as searching around the zone for any other alga not present in the quadrats in order to truly cover all species available to herbivores. The most abundant basal resources, which were further considered for analyses of quality and stable isotopes (4 replicates from the pooled material) or feeding assays (n = 5–10 replicates; see Fig. 4), were the leaves of the seagrass *Posidonia* (pooled second-outermost leaves from 6 shoots), their epiphytes (removed from the seagrass leaves with a glass slide), the four most abundant algae (*Flabellia petiolata*, *Halopteris scoparia*, *Jania rubens*, and *Peyssonnelia* spp.), and the relatively abundant *Cladophora prolifera* (here named *Cladophora*). Other resources that were scarce in some meadows but present in urchin guts (*Asparagopsis armata*, *Dictyota* spp., and *Cladophora* spp.) were only collected for SIA (with 4, 3 and 1–2 replicates, respectively).

In each site, 10 sea urchins and 3 wrasses were caught (no wrasse was caught at the north-vent), euthanized, measured and dissected for extraction of guts and muscle samples. They were adult individuals of similar size (mean ± SD; sea urchins: 5.1 ± 0.5 cm in diameter without spines; wrasses: 13.1 ± 0.7 cm in total length). A sample of white muscle was collected under the dorsal fin from wrasses, while the Aristotle’s lantern muscle was collected from sea urchins. Samples were freeze-dried and reduced to fine powder for laboratory analyses.

### Consumer feeding

Carbon and nitrogen stable isotopes (plus C and N content) of producers and consumers were analysed with an isotope ratio mass spectrometer (ThermoFinnigan DeltaPlus) coupled to an elemental analyser (ThermoFinnigan FlashEA1112). They were expressed in delta notation, as parts per thousand deviation from the corresponding standard reference materials (Vienna Pee Dee Belemnite and atmospheric N_2_). Analytical precision based on the deviation from internal standards was ±0.15‰. Samples of epiphytes and calcified algae were acidified (HCl 2 N) prior to δ^13^C analysis to remove carbonates. Phosphorus (P) content was determined using inductively coupled plasma optical emission spectrophotometry (Perkin Elmer, Optima 8000), after acid (HNO_3_/H_2_O_2_) digestion. To obtain enough material for P analysis, samples of sea urchin muscle were pooled (vents n = 3; off-vent n = 4). CNP ratios were calculated on a molar basis.

Isotopic data of sea urchin populations (n = 10 for each site) were used to estimate the Layman metrics of trophic structure^48^ and the Standard Ellipse Areas using the SIBER package v2.0.2 in R^49^. Specifically, the δ^15^N range (NR) and δ^13^C range (CR) are the Euclidean distance between the highest and the lowest δ^15^N and δ^13^C values. NR represents the trophic length or the degree of omnivory (high NR = more individuals feeding at different trophic levels) and CR the diversity of basal resources exploited (high CR = more carbon sources). The mean distance to centroid (CD) is the average Euclidean distance of each individual to the mean δ^15^N and δ^13^C values of all individuals in the population and represents the trophic diversity. The mean nearest neighbour distance (NND) measures the density of individuals’ packing or trophic redundancy. The standard deviation of the nearest neighbour distance (SDNND; less influenced than NND by sample size) measures the evenness of the population packing (low NND and SDNND = individuals in a population with similar trophic habits). Metrics of urchin populations were bootstrapped (n  =  10000; indicated with a subscript ‘b’) to allow comparison among populations^50^. We estimated the isotopic niche width of urchin populations from the Bayesian Standard Ellipse Area (SEAb; with SIBER default priors and Monte Carlo options) and the corrected Standard Ellipse Area (SEAc, corrected for small sample size). Differences between sites in bootstrapped metrics and Bayesian ellipse areas were tested regarding their dissimilarity (p > 0.95) using the probability test proposed in the SIBER package. Due to the low replication of predator populations (n < 5), we were only able to estimate single values of Layman metrics using SIBER (but not bootstrapped metrics or standard ellipses), whose differences cannot be tested for statistical significance. Furthermore, we examined differences between sites in the position of the niche centroid using a bivariate permutational analysis of variance (PERMANOVA) on δ^13^C and δ^15^N values grouped by site and separately tested for sea urchins (3 sites: north-, south- and off-vent) and wrasses (2 sites: south- and off-vent).

Diet composition was estimated as the proportion of resources/preys in consumer guts (% of horizontal surface covered) that were identified and quantified under a stereomicroscope. Labrid fishes feed on sea urchins by entirely swallowing small recruits (<1 cm) or by eating soft meat after opening the test of larger juveniles that they are able to bite (1–3 cm)^17^. This limited the recognition of sea urchins in wrasse gut to rests of long-lasting calcareous structures (Aristotele lantern, skeleton or spines) of small recruits that were entirely swallowed. Very deteriorate pieces of such tiny rests were likely included in the category ‘Other’. Taking that into account, a MixSIAR model in R^51^ was used to estimate, and further confirm, the role of sea urchins in wrasse diet (Supplementary Table S2). Sea urchin diet was obtained from Nogueira *et al*.^24^. Similarity percentage analyses (SIMPER) were used to identify food items in each consumer diet with higher and consistent contribution to the between-site dissimilarity. A consistent contribution (namely, higher than variation) was arbitrarily defined as that with a mean dissimilarity to standard deviation ratio> 1.5.

We obtained consumer trophic positions (TP_c_) from diet data (excluding ‘other material’) by using an equation modified from Vander Zanden & Rasmussen^52^ as follows: TP_c_ = ∑(C_i_.TP_i_) + 1, where C_i_ = contribution of the prey i to the diet, and TP_i_ = trophic position of the food item i obtained as detailed in Supplementary Table S3.

We also estimated consumer trophic positions from SIA data using the equation proposed by Post^53^: TP_c_ = [(δ^15^N_c_ − δ^15^N_b_)/∆δ^15^N] + λ, where subscript c and b respectively refers to the consumer and to the baseline reference species; ∆δ^15^N is the expected enrichment in δ^15^N between successive trophic levels; and λ is the trophic position of the species chosen as baseline. The baseline for sea urchins, with a trophic level λ = 1, was the site-specific mean δ^15^N value of the main basal resources (those with abundance > 2% and a total contribution of ca. 90% to the diet, excluding epiphytes). The baseline for wrasses, with a trophic level λ = 2, was obtained from primary consumers showing the lowest δ^15^N values (site-specific values of other benthic invertebrates obtained from Ricevuto *et al*.^43^ in the same sites and season). The isotopic fractionation considered was ∆δ^15^N = 2.52‰ for the herbivore and 3.23‰ for the predator^54^.

### Resource quality and palatability to herbivore

Indicators of nutritional (CNP contents and ratios obtained as described above, carbohydrates) and chemical quality (total phenolics) were measured on the most abundant basal resources available to herbivores. Non-structural carbohydrates were measured using the phenol-sulfuric acid colorimetric method^55^ with glucose as standard, after extraction of soluble sugars (mostly sucrose) in hot ethanol and enzymatic conversion of starch to glucose equivalents. Total phenolics were extracted with 80% methanol for 24 h and determined with spectrophotometer after a Folin-Ciocalteu assay using caffeic acid as standard (modified from Bolser *et al*.^56^). Only 3 replicates were available for P, phenolics and sucrose/starch analyses of *Cladophora* at the off-vent. Five replicates of *Posidonia* and *Peyssonnelia* were analysed per site for phenolic content because a high variability among replicates was detected.

The carbonate content of calcareous resources (epiphytes, *Jania* and *Peyssonnelia*) was quantified as structural defence by the loss of dry weight after decalcification of samples with HCl (2 N). Structural traits of seagrass leaves were measured in the second-outermost (fully-developed) leaf of 14 of those shoots used to estimate fish herbivory. Leaf width and thickness were measured at 3.5 cm above the sheath junction with an electronic digital thickness gauge (Miyare, precision ± 0.01 mm), and then multiplied to calculate leaf cross-sectional area. Three of the 14 replicates from the north-vent were lost during sample processing.

We conducted feeding preference assays to investigate whether resources are more or less palatable to sea urchins in response to OA. Assays were conducted in 24 L aquaria with aerated seawater offering comparable amounts of off-vent and vent material of each resource to an adult sea urchin (5.4 ± 0.5 cm diameter, n = 42). A relatively old tissue of *Posidonia* leaves was used (ca. 8 cm^2^ of the second-outermost leaf, avoiding necrotic apices), as this is preferentially eaten over young tissue by this sea urchin species^25^. Fresh weight offered varied depending on the resource (ca. 0.3 g *Posidonia*, *Flabellia*, and *Peyssonnelia*; ca. 0.5 g *Halopteris*; and ca. 1 g *Jania*). For each resource, 5 to 10 assay replicates were set up, as well as 3 to 4 control replicates with resource material and no sea urchin to correct consumption for autogenic changes during assays. Prior to the start of the assays, sea urchins were acclimatized and starved for 24 hours. Assays were ended when 50% of any resource material was consumed. Consumption of each resource (% of the initial wet mass offered that was consumed) was calculated as [(H_i_ × C_f_/C_i_)−H_f_] × 100/H_i_, where H_i_ and H_f_ are initial and final wet masses of the resource and C_i_ and C_f_ the mean initial and final wet masses of the corresponding autogenic controls. All assays were performed in accordance with appropriate guidelines and regulations.

To examine the significance of the preference, we estimated the effect size using Hedges’ d (the difference in consumption means between off-vent and vent weighted by the sample size and the pooled standard deviation) and its confidence intervals (CI)^57^, since our data followed a normal distribution. When CI did not include zero, the effect size was significant at the 5% level.

### Habitat structure

The structural complexity of the seagrass habitat was estimated as the heights of the leaf canopy and of the unburied rhizome layer in 25 random areas of 20 cm × 20 cm per site. The canopy height was measured from the base to the top of the leaves ignoring the 20% of the tallest ones (modified from Short & Coles^58^). The unburied level of the rhizome layer was measured as the distance between the leaf base and the sediment surface by inserting a ruler into the three-dimensional net formed by the overlapping vertical and horizontal rhizomes^59^. Both variables, together with seagrass shoot density, are related to refuge provision of *Posidonia* meadows to sea urchins^60^. Corrected indicators of refuge provision were obtained by multiplying each replicated valued of canopy and rhizome heights by the number of shoots in the sampling area using shoot density data at the same sites and depth obtained from the literature (mean value per site).

Fish (salema) herbivory is known to mediate indirect interactions operating at local spatial scales by reducing the seagrass canopy height and the refuge provision, with a subsequent increase in the predation of sea urchins^61^. To take this into account, we quantified the percentage of leaves with fish bite marks in one shoot within each of the 25 above-mentioned areas. Bite marks of salemas were easily differentiated due to their characteristic half-moon shape^62^.

### Consumer abundance

We quantified consumer abundances in each site using visual censuses along five (5 m length × 2 m width) transects for sea urchin and six (10 m length × 4 m width) transects for fish. Consumer abundances were expressed as number of individuals per squared meter. Whilst transects in each meadow were surveyed during the morning in a single day, results were consistent to divers’ observations at different times on several days. *P. lividus* was the only urchin species found. Adult sea urchins found in transects were part of those collected for SIA, diet analysis and feeding assays.

### Other data analyses

Differences between sites were analysed separately for each variable using PERMANOVA, since most of our data did not conform to parametric test assumptions. We used one-way PERMANOVAs to examine differences among sites (fixed factor, 3 levels) in seagrass canopy height, rhizome layer, fish herbivory, and leaf thickness, width, and cross-sectional area, as well as in consumer isotopic signatures, elemental contents and ratios, trophic position and diet composition analysed separately for urchins and wrasses (3 and 2 levels, respectively, as no wrasse was caught at the north-vent). Two-way PERMANOVAs were used to examine differences between sites (3 levels) and resources in isotopic signatures (resources: 10 levels), quality traits and CNP contents or ratios (resources: 3 levels for calcification and 7 levels for the rest), as well as between sites and consumers (2 levels) in their abundances. We calculated Euclidean distances from untransformed data and used 9999 unrestricted permutations of raw data to perform the tests, except in consumer abundances and fish herbivory (both (x + 0.001)-transformed data) or consumer diet (untransformed data) for which Bray-Curtis distances were used. When significant differences were found, pairwise comparisons were performed. When the number of possible permutations was low (<100) in any PERMANOVA test or pairwise comparison, the Monte Carlo p-value was used instead of the permutation p-value^63^.

## Supplementary information

Supplementary Information.

## Data Availability

The data that support the findings of this study are included in the figures of the article and its supplementary information. The raw data used to generate the figures and the R codes used to analyse them are available from the corresponding author upon reasonable request.
