# Supplementary material for: Resistance of seagrass habitats to ocean acidification via altered interactions in a tri-trophic chain
Source: Sci Rep. 2020 Mar 20;10:5103. doi: 10.1038/s41598-020-61753-1 (PMC7083920; doi:10.1038/s41598-020-61753-1)
Supplement: Supplementary file 1 — Supplementary Information. [file 41598_2020_61753_MOESM1_ESM.pdf]

Supplementary Information for

Resistance of seagrass habitats to ocean acidification via altered interactions in a tri-trophic chain

Martínez-Crego B, Vizzini S, Califano G, Massa-Gallucci A, Andolina C, Gambi MC, Santos R

Supplementary Table S1. Seawater chemistry. Mean (± SD, n = 5) seawater chemical parameters in the studied off-vent and vent sites. Seawater pH and temperature were measured at the time of sampling using a Multimeter 340 WTW (Weilheim, Germany; corrected for temperature and calibrated using NBS buffers). Total alkalinity observed in this sampling season averages 2563 µmol kg^{-1} at ambient conditions and 2560 µmol kg^{-1} at vent sites (measured using the titration method on discrete water samples and checking accuracy with certified reference material provided by Scripps Institution of Oceanography, La Jolla, CA) and salinity 37.9 and 37.8, respectively[1,2]. These data were used to estimate pCO_2 and carbonate chemistry using the CO2SYS program v2.1 (modified by Pierrot et al.[3]) with the constants of Mehrbach refit by Dickson & Millero[4]).

Site	T (ºC)	pH	pCO_2 (µatm)	HCO_3^- (µmol kg^{-1})	CO_3^{2-}(µmol kg^{-1})
Off-vent	23.0 ± 0.04	8.10 ± 0.01	543 ± 13	2063 ± 7.9	205 ± 3.2
North-vent	22.8 ± 0.09	7.78 ± 0.02	1262 ± 74	2296 ± 13	109 ± 5.1
South-vent	22.7 ± 0.05	7.80 ± 0.04	1176 ± 108	2281 ± 20	115 ± 8.3

Supplementary Table S2. Contribution of food sources to wrasse diet estimated through Bayesian mixing models using the MixSIAR package in R. Contributions are expressed as mean (± SD) with the 5th and 95th percentiles of the posterior source contributions. In the model, we considered four main food sources (*Posidonia*, epiphytes, the sea urchin *Paracentrotus lividus*, and other invertebrates) and trophic enrichment factors of 0.4 ± 1.3‰ for $\delta^{13}C$ and 3.4 ± 1.0‰ for $\delta^{15}N$ according to Post[5].

	**Off-vent**			**South-vent**		
**Food source**	**Mean ± SD**	**5%**	**95%**	**Mean ± SD**	**5%**	**95%**
Posidonia	0.23 ± 0.14	0.03	0.46	0.23 ± 0.14	0.03	0.47
Epiphytes	0.25 ± 0.17	0.02	0.55	0.25 ± 0.17	0.02	0.55
Paracentrotus	0.27 ± 0.18	0.03	0.59	0.27 ± 0.18	0.03	0.59
Other invertebrates	0.25 ± 0.16	0.03	0.53	0.25 ± 0.16	0.02	0.53

Supplementary Table S3. Trophic level (TL) of food sources used to calculate the diet-based trophic positions of consumers. * TL as in Vizzini et al.[6]. Deposit and filter feeders TL=2.2 (also Foraminifera that usually grazes on diatoms but are also deposit feeders); animals showing diverse feeding types (Nematoda, Sipunculida, Pantopoda) TL=2.5 (as for Polychaeta), and mainly carnivores TL=2.8 according to http://www.sealifebase.org.

Food source	TL
Producers	1.0
Epiphytes	1.8
Bivalvia	2.2
Gastropoda*	2.4
Decapoda*	2.8
Cumacea	2.2
Amphipoda*	2.3
Copepoda	2.8
Isopoda*	2.0
Ostracoda	2.2
Tanaidacea*	2.3
Acari	2.8
Pantopoda	2.5
Foraminifera	2.2
NC Polychaeta*	2.5
Nematoda	2.5
Sipunculida	2.5

Supplementary Table S4. Trophic structure of sea urchin populations at off-vent and vent sites.

(a) Results of boostrapped Layman metrics (with relative credible intervals) and Bayesian ellipse areas (SEAb with mode and SEAc) calculated using SIBER, which were compared for significant dissimilarity ($p > 0.95$) between sites using the probability tests proposed in the SIBER package.

	off-vent	north-vent	south-vent
**SEAc**	1.3	1.8	2.1
**SEAb**	1.1<br>(0.68 - 2.5)	1.5<br>(0.96 - 3.4)	1.8<br>(1.1 - 4.1)
**NR_b**	4.1<br>(0.63 - 4.1)	4.1<br>(2.2 - 4.1)	4.2<br>(1.1 - 4.2)
**CR_b**	0.91<br>(0.55 - 0.91)	1.2<br>(0.12 - 1.22)	1.6<br>(0.47 - 1.6)
**CD_b**	0.87<br>(0.29 - 1.6)	1.1<br>(0.65 - 1.58)	1.1<br>(0.46 - 1.7)
**NND_b**	0.17<br>(0.04 - 0.65)	0.31<br>(0.07 - 0.60)	0.27<br>(0.04 - 0.74)
**$SDNND_b$**	0.24<br>(0.09 - 1.1)	0.38<br>(0.17 - 0.84)	0.46<br>(0.06 - 1.2)

	off vs. north	off vs. south	north vs. south
**SEAb**	0.76	0.87	0.34
**NR_b**	0.53	0.68	0.43
**CR_b**	0.58	0.92	0.13
**CD_b**	0.78	0.70	0.59
**NND_b**	0.56	0.60	0.43
**$SDNND_b$**	0.56	0.59	0.33

(b) Results from one-way PERMANOVA examining differences between sites in the position of the isotopic niche (= centroid in the δ^{13}C- δ^{15}N isotopic space).

Source of variation	df	MS	Pseudo-F	p-value (perm)	Unique perms	Pairwise comparison
Site	2	19	26	0.001	999	north- > south- > off-vent
Residual	27	0.73				

Supplementary Table S5. Trophic structure of wrasse populations at off-vent and vent sites.

(a) Results of Layman metrics (single values) calculated using SIBER.

	off-vent	south-vent
**NR**	0.31	1.74
**CR**	0.93	0.20
**CD**	0.37	0.74
**NND**	0.46	0.70
**SDNND**	0.14	0.75

(b) Results from one-way PERMANOVA examining differences between sites in the position of the isotopic niche (= centroid in the δ^{13}C- δ^{15}N isotopic space). The Monte Carlo p-value was used due to the low the number of possible permutations (`Unique perms´).

Source of variation	df	MS	Pseudo-F	p-value (MC)	Unique perms
Site	1	4.7	3.6	0.09	10
Residual	4	1.3			

Supplementary Table S6. **Comparison of isotopic signatures of resources at off-vent and vent sites.** Results from two-way PERMANOVA examining differences in isotopic signatures between resources and sites. Resources are indicated in pairwise comparisons as follows: Asp, *Asparagopsis armata*; Clado, *Cladophora prolifera*; Cla, *Cladophora* spp.; Dic, *Dictyota* spp.; Epi, Epiphytes; Fla, *Flabellia petiolata*; Halo, *Halopteris scoparia*; Jan, *Jania rubens*; Pey, *Peyssonnelia* spp.; Posi: *Posidonia oceanica*.

Variable	Source of variation	df	MS	Pseudo-F	p-value (perm)	Unique perms
$\delta^{13}C$	Resource	9	385	1399	0.0001	9950
	Site	2	5.9	21	0.0001	9947
	Resource x Site	15	6.6	24	0.0001	9912
	Residual	70	0.28			
$\delta^{15}N$	Resource	9	5.3	204	0.0001	9936
	Site	2	46	1778	0.0001	9958
	Resource x Site	15	0.46	18	0.0001	9922
	Residual	70	0.03			

Variable	Pairwise comparison	
$\delta^{13}C$	Resource x Site (Site)	Posi: S ~ off > N; Halo: off > N > S; Fla: off ~N > S; Asp: N > off; Epi: S > off, N ~ S, N ~ off; Dic: N > S; Pey: N > S (Clado and Jan ns; Cla not tested)
$\delta^{15}N$	Resource x Site (Site)	Posi, Halo, Fla, Clado, Pey, Epi: N > S > off; Asp, Jan: N > off, Dic: N > S (Cla not tested)

Supplementary Table S7. **Comparison of consumer isotopic signatures at off-vent and vent sites.** Results from one-way PERMANOVA examining differences in isotopic signatures between sites for each consumer separately.

Consumer	Variable	Source of variation	df	MS	Pseudo-F	p-value (perm/MC)	Unique perms	Pairwise comparisons
P. lividus	$\delta^{13}C$	Site	2	26	15	0.0001	9961	off ~ south > north (off-south p=0.065)
		Residual	27	1.7				
	$\delta^{15}N$	Site	2	8.8	49	0.0001		north > south > off
		Residual	27	0.18				
S. tinca	$\delta^{13}C$	Site	1	2.6	22	0.009	10	south > off
		Residual	4	0.12				
	$\delta^{15}N$	Site	1	0.20	0.41	0.55	10	-
		Residual	4	0.47				

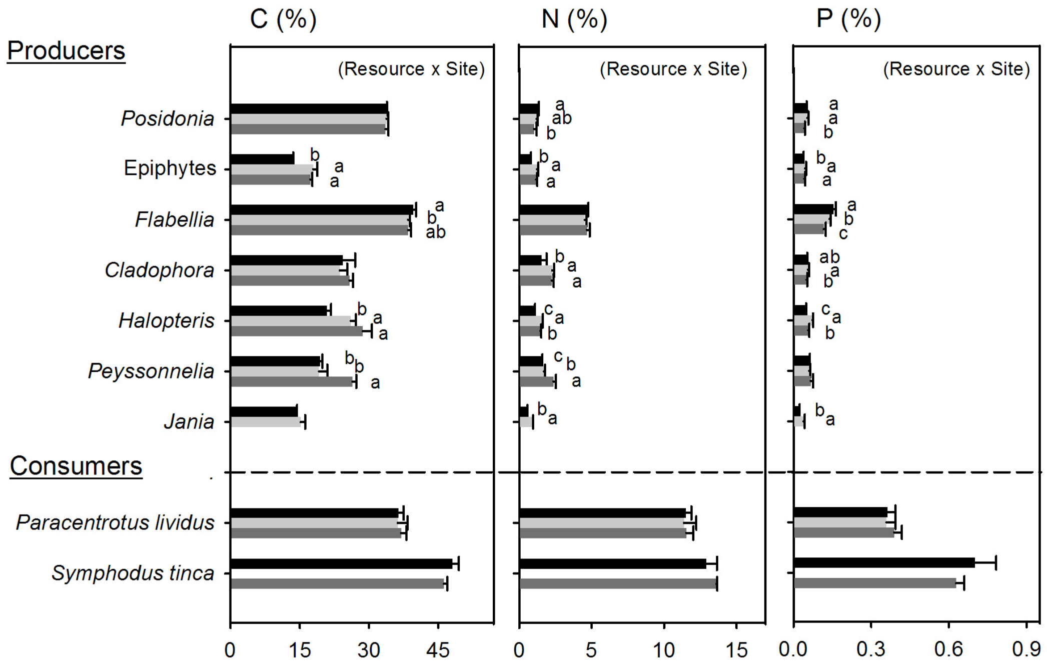

Supplementary Figure S1. Nutrient contents of resources and consumers at off-vent and vent sites (mean ± SD). Significant effects on resources from two-way PERMANOVA are shown within the parenthesis. Different letters next to bars denote statistically significant differences between off-vent (black) and vent (light grey: north-vent, dark grey: south-vent) sites based on post hoc comparisons.

Supplementary Table S8. **Comparison of consumer stoichiometry at off-vent and vent sites.** Results from one-way PERMANOVA examining differences between sites in nutrient contents and C:N:P stoichiometry of consumers.

Consumer	Variable	Source of variation	df	MS	Pseudo-F	p-value (perm/MC)	Unique perms	Pairwise comparisons
P. lividus	C	Site	2	1.6	0.69	0.54	9960	-
		Residual	27	2.3				
	N	Site	2	0.07	0.19	0.86	9956	-
		Residual	27	0.38				
	P	Site	2	0.0008	0.84	0.46	2059	-
		Residual	7	0.0010				
	CN	Site	2	0.0045	1.1	0.37	9944	-
		Residual	27	0.0041				
	C:P	Site	2	226	0.60	0.56	2076	-
		Residual	7	379				
	N:P	Site	2	22	0.93	0.42	2080	-
		Residual	7	24				

Consumer	Variable	Source of variation	df	MS	Pseudo-F	p-value (perm/MC)	Unique perms	Pairwise comparisons
S. tinca	C	Site	1	5.4	4.2	0.11	10	-
		Residual	4	1.3				
	N	Site	1	0.69	2.5	0.18	10	-
		Residual	4	0.27				
	P	Site	1	0.008	2.0	0.23	10	-
		Residual	4	0.004				
	CN	Site	1	0.23	3.3	0.14	10	-
		Residual	4	0.07				
	C:P	Site	1	184	0.50	0.52	10	-
		Residual	4	371				
	N:P	Site	1	73	13	0.02	10	south > off
		Residual	4	5.5				

Supplementary Table S9. **Trophic position of consumers.** Results from one-way PERMANOVA examining differences in consumer trophic levels between sites for each consumer separately.

	Method	Source of Variation	df	MS	Pseudo-F	p-value (perm/MC)	Unique perms	Pairwise comparisons
P. lividus	Diet-based	Site	2	0.01	8.8	0.001	9927	off > north ~ south
		Residual	26	0.001				
	SIA-based	Site	2	0.11	3.8	0.03	9952	off > north
		Residual	27	0.03				
S. tinca	Diet-based	Site	1	0.00002	0.001	0.98	10	-
		Residual	4	0.0				
	SIA-based	Site	1	1.7	37	0.004	10	off > south
		Residual	4	0.05				

Supplementary Table S10. Consumer (herbivore) diet. Results of SIMPER analysis showing the abundance of food items in sea urchin diet (% cover in the gut content; mean ± SD) and their contribution to the dissimilarity in diet composition between sites. Calcareous epiphytes identified in urchin diet were dominated by crustose coralline, but also may contain animal material such as bryozoans and polychaeta tubes that was not possible to identify separately.

Total food diversity in sea urchin diet comprised 14 food items (*Posidonia*, calcareous epiphytes, and 12 algal taxa), including 13 taxa in both off-vent and north-vent and 12 taxa south-vent. No animal preys were present in the diet of this strict herbivore. The

SIMPER analysis showed 84%, 65% and 66% similarity within replicates in the urchin diet at off-vent, north-vent and south-vent, respectively.

Group	Dissimilarity = 40.05	Abundance off-vent	Abundance north-vent	Diss/SD	Contribution (%)	Cumulative (%)
Seagrass	*Posidonia oceanica*	68.6 ± 8.0	41.2 ± 19.6	1.5	35.5	35.5
Green algae	*Flabellia petiolata*	2.0 ± 3.0	14.6 ± 8.8	1.6	16.3	51.8
Green algae	*Cladophora prolifera*	0.6 ± 0.8	11.6 ± 6.0	1.9	13.7	65.5
Epiphytes	Calcareous epiphytes	15.3 ± 3.8	8.9 ± 2.5	1.6	8.2	73.7
Other	Other unidentified material	4.0 ± 4.0	5.8 ± 4.6	0.98	5.3	79.0
Brown algae	*Halopteris scoparia*	1.2 ± 1.2	4.5 ± 4.1	1.3	5.1	84.1
Red algae	Peyssonnelia spp.	4.2 ± 2.4	5.7 ± 4.1	1.6	5.0	89.1
Red algae	*Jania rubens*	2.1 ± 3.6	3.2 ± 4.5	0.81	4.4	93.5
Green algae	*Derbesia* spp.	0.03 ± 0.05	3.1 ± 9.1	0.36	3.8	97.3
Green algae	*Cladophora* spp.	1.2 ± 1.3	1.2 ± 1.0	1.2	1.6	98.8
Red algae	*Acrodiscus* cf. *vidovichii*	0.5 ± 1.7	0.03 ± 0.10	0.35	0.7	99.6
Red algae	*Polysiphonia* spp.	0.2 ± 0.6	Absent	0.33	0.3	99.8
Red algae	*Asparagopsis* spp.	0.05 ± 0.08	0.09 ± 0.09	1.1	0.1	99.91
Brown algae	*Dictyota* spp.	0.03 ± 0.09	0.01 ± 0.03	0.43	0.05	99.96
Green algae	*Ulva* spp.	Absent	0.03 ± 0.10	0.35	0.04	100
	Dissimilarity = 34.74	Abundance off-vent	Abundance south-vent	Diss/SD	Contribution (%)	Cumulative (%)
Seagrass	*Posidonia oceanica*	68.6 ± 8.0	49.2 ± 17.8	1.5	32.2	32.2
Green algae	*Cladophora prolifera*	0.6 ± 0.8	16.0 ± 11.2	1.4	22.2	54.3
Epiphytes	Calcareous epiphytes	15.3 ± 3.8	8.4 ± 5.3	1.8	11.6	65.9
Green algae	*Flabellia petiolata*	2.0 ± 3.0	8.0 ± 9.6	0.8	10.5	76.4
Red algae	*Jania rubens*	2.1 ± 3.6	4.1 ± 4.5	0.9	5.7	82.1
Red algae	*Peyssonnelia* spp.	4.2 ± 2.4	4.6 ± 3.2	1.4	4.5	86.6
Brown algae	*Halopteris scoparia*	1.2 ± 1.2	3.6 ± 3.0	1.1	4.2	90.7
Other	Other unidentified material	4.0 ± 4.0	2.6 ± 1.2	0.7	3.4	94.1
Green algae	*Cladophora* spp.	1.2 ± 1.3	1.8 ± 1.6	1.2	2.3	96.4
Red algae	Asparagopsis spp.	0.05 ± 0.08	0.9 ± 0.8	1.2	1.3	97.7
Red algae	*Acrodiscus* cf. *vidovichii*	0.5 ± 1.7	0.04 ± 0.10	0.4	0.8	98.5
Brown algae	*Dictyota* spp.	0.03 ± 0.09	0.5 ± 1.6	0.4	0.8	99.3
Red algae	*Polysiphonia* spp.	0.2 ± 0.6	0.3 ± 0.9	0.5	0.7	100.0
Green algae	*Derbesia* spp.	0.03 ± 0.05	Absent	0.7	0.04	100
	Dissimilarity = 35.14	Abundance north-vent	Abundance south-vent	Diss/SD	Contribution (%)	Cumulative (%)
Seagrass	*Posidonia oceanica*	41.2 ± 19.6	49.2 ± 17.8	1.41	30.5	30.5
Green algae	*Flabellia petiolata*	14.6 ± 8.8	8.0 ± 9.6	1.5	16.6	47.1
Green algae	*Cladophora prolifera*	11.6 ± 6.0	16.0 ± 11.2	1.31	14.5	61.6
Epiphytes	Calcareous epiphytes	8.9 ± 2.5	8.4 ± 5.3	1.35	6.4	68.0
Red algae	*Jania rubens*	3.2 ± 4.5	4.1 ± 4.5	0.99	6.1	74.1
Red algae	*Peyssonnelia* spp.	5.7 ± 4.1	4.6 ± 3.2	1.44	5.9	80.0
Brown algae	*Halopteris scoparia*	4.5 ± 4.1	3.6 ± 3.0	1.41	5.6	85.6
Other	Other unidentified material	5.8 ± 4.6	2.6 ± 1.2	0.95	5.4	91.0
Green algae	*Derbesia* spp.	3.1 ± 9.1	Absent	0.36	4.4	95.4
Green algae	Cladophora spp.	1.2 ± 1.0	1.8 ± 1.6	1.17	2.1	97.4
Red algae	*Asparagopsis* spp.	0.09 ± 0.09	0.9 ± 0.8	1.19	1.3	98.7
Brown algae	*Dictyota* spp.	0.01 ± 0.03	0.5 ± 1.6	0.34	0.7	99.4
Red algae	*Polysiphonia* spp.	Absent	0.3 ± 0.9	0.33	0.4	99.9
Red algae	*Acrodiscus* cf. *vidovichii*	0.03 ± 0.10	0.04 ± 0.10	0.56	0.09	99.95
Green algae	*Ulva* spp.	0.03 ± 0.10	Absent	0.35	0.05	100

Supplementary Table S11. Consumer (predator) diet. Results of SIMPER analysis showing the abundance of food items in wrasse diet (% cover in the gut content; mean ± SD) and their contribution to the dissimilarity in diet composition between sites. Grouping abbreviations are as follows: NC invert = Non-calcified benthic invertebrates; Other LC invert = Less calcified benthic invertebrates other than amphipods; Other HC invert = Heavily calcified benthic invertebrates other than gastropods; Other = Other unidentified material.

Wrasse diet comprised mostly benthic invertebrates (87.3 ± 3.2 % off-vent and 84.6 % ± 12.7 % south-vent), but also *Posidonia* and algae (5.8 ± 1.4 % off-vent and 11.8 % ± 12.0 % south-vent) and other unidentified material (the rest). The SIMPER analysis showed 43% and 54% similarity within replicates in the wrasse diet at off-vent and south-vent, respectively. Only gastropods and foraminifera (with a high contribution), as well as Ostracods (with a low contribution), consistently contributed (Diss/SD > 1.5) to the between-site dissimilarity.

Group	Dissimilarity=58.0	Abundance off-vent	Abundance south-vent	Diss/SD	Contribution (%)	Cumulative (%)
Gastropods	Gastropoda	12.9 ± 11.1	28.5 ± 5.6	1.7	13.9	13.9
Amphipods	Amphipoda	8.0 ± 1.4	22.8 ± 20.0	0.9	12.9	26.8
Other LC invert	Isopoda	12.7 ± 20.6	0.6 ± 1.0	0.7	10.8	37.6
NC invert	Sipunculida	11.4 ± 19.7	1.9 ± 3.3	0.8	10.3	47.9
Other HC invert	Foraminifera	16.0 ± 2.9	6.4 ± 6.8	1.7	8.5	56.5
Other HC invert	Bivalvia	9.2 ± 14.1	1.3 ± 1.1	0.8	7.5	64.0
Other LC invert	Decapoda	Absent	7.8 ± 8.0	1.1	6.7	70.7
NC invert	NC Polychaeta	7.8 ± 8.6	4.8 ± 2.2	1.3	5.5	76.2
Seagrass	*Posidonia*	3.5 ± 1.6	6.3 ± 9.4	1.1	5.4	81.6
Other	Other material	6.9 ± 3.	3.6 ± 0.7	1.5	3.2	84.8
Other LC invert	Copepoda	4.3 ± 2.1	4.3 ± 3.8	1.5	2.6	87.4
Algae	Chlorophyta n.i.	Absent	2.4 ± 2.2	1.3	2.0	89.4
Other LC invert	Ostracoda	Absent	2.3 ± 0.5	4.8	1.9	91.4
Other LC invert	Tanaidacea	1.8 ± 1.6	2.1 ± 1.9	1.2	1.4	92.8
Algae	Calc. articulated Rhodophyta	1.1 ± 1.8	1.2 ± 2.1	0.9	1.3	94.1
Algae	NC corticated Rhodophyta	Absent	1.4 ± 2.5	0.7	1.2	95.3
Other LC invert	Cumacea	1.3 ± 1.2	Absent	1.3	1.1	96.4
Other LC invert	Pantopoda	Absent	1.2 ± 2.1	0.7	1.0	97.4
Algae	NC filamentous Rhodophyta	1.3 ± 1.2	0.6 ± 1.0	1.2	1.0	98.4
Other LC invert	Acari	1.3 ± 1.2	0.6 ± 1.0	1.2	1.0	99.4
NC invert	Nematoda	0.7 ± 1.2	Absent	0.7	0.6	100

Supplementary Table S12. Comparison of consumer diet at off-vent and vent sites. Results from one-way PERMANOVA using Bray-Curtis distances that examined differences in diet composition (% cover of food items) between sites for each consumer separately.

	Source of variation	df	MS	Pseudo-F	p-value (perm/MC)	Unique perms	Pairwise comparisons
P. lividus	Site	2	3375	6.9	0.0001	9943	off ≠ north ~ south
	Residual	26	489				
S. tinca	Site	1	2654	2.0	0.15	10	-
	Residual	4	1347				

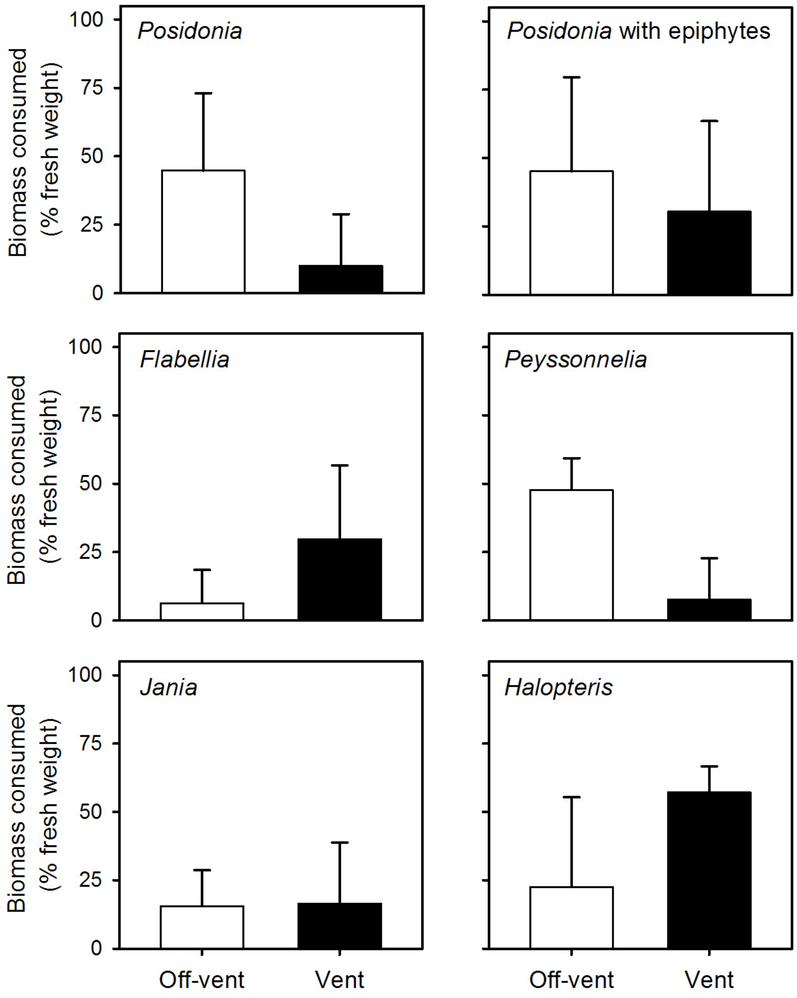

Supplementary Figure S2. **Palatability to herbivores of resources grown at off-vent and vent sites**. Consumption (mean ± SD % of the of the wet mass offered that was consumed) in feeding assays examining the preference by sea urchins between vent and off-vent material of 6 different resources independently offered.

Supplementary Table S13. Comparison of the seagrass structural quality as food at off-vent and vent sites. Results from one-way PERMANOVA examining differences between sites in structural leaf traits of the seagrass.

	Source of variation	df	MS	Pseudo -F	p-value (perm)	Unique perms	Pairwise comparisons
Width	Site	2	0.65	1.4	0.262	2664	-
	Residual	36	0.47				
Thickness	Site	2	0.03	6.4	0.004	3236	north ~ south > off
	Residual	36	0.004				
Cross-sectional area	Site	2	3.6	5.9	0.006	9947	north ~ south > off
	Residual	36	0.61				

Supplementary Table S14. **Comparison of the resource quality as food at off-vent and vent sites.** Results from two-way PERMANOVA examining differences between resources and sites in nutritional and chemical quality.

Variable	Source of variation	df	MS	Pseudo-F	p-value (perm)	Unique perms
C	Resource	6	816	684	0.0001	9948
	Site	2	62	52	0.0001	9946
	Resource x Site	11	18	15	0.0001	9916
	Residual	60	1.2			
N	Resource	6	19	1208	0.0001	9934
	Site	2	0.92	58	0.0001	9946
	Resource x Site	11	0.24	15	0.0001	9934
	Residual	60	0.02			
P	Resource	6	0.01	634	0.0001	9952
	Site	2	0.001	28	0.0001	9942
	Resource x Site	11	0.0004	17	0.0001	9930
	Residual	59	0.00002			
C:N	Resource	6	753	146	0.0001	9948
	Site	2	95	18	0.0001	9953
	Resource x Site	11	40	7.7	0.0001	9936
	Residual	60	5.2			
C:P	Resource	6	1457600	155	0.0001	9953
	Site	2	490150	52	0.0001	9938
	Resource x Site	11	68986	7.3	0.0001	9935
	Residual	59	9400			
N:P	Resource	6	1963	80	0.0001	9952
	Site	2	1158	47	0.0001	9944
	Resource x Site	11	140	5.7	0.0001	9935
	Residual	59	25			
Phenolics	Resource	6	30	664	0.0001	9958
	Site	2	0.78	17	0.0001	9945

Variable	Source of variation	df	MS	Pseudo-F	p-value (perm)	Unique perms
	Resource x Site	11	0.16	3.6	0.001	9952
	Residual	65	0.05			
Sucrose	Resource	6	67	703	0.0001	9951
	Site	2	4.2	44	0.0001	9951
	Resource x Site	11	0.9	9.2	0.0001	9916
	Residual	59	0.10			
Starch	Resource	6	50	262	0.0001	9935
	Site	2	20	105	0.0001	9959
	Resource x Site	11	12	61	0.0001	9938
	Residual	59	0.19			
Calcification	Resource	2	1402	113	0.0001	9962
	Site	2	44.3	3.6	0.050	9957
	Resource x Site	3	16.3	1.3	0.297	9956
	Residual	24	12.4			

Supplementary Table S15. Comparison of consumer abundances at off-vent and vent sites. Results from two-way PERMANOVA examining differences in abundances (# of individuals m^{-2}) between consumers and sites. Tests were run using Bray-Curtis distances on (x+0.001)-transformed data. Consumers are indicated as follows: *Paracentrotus lividus* (Pl), *Symphodus tinca* (St).

Source of variation	df	MS	Pseudo-F	p-value (perm)	Unique perms	Pairwise comparisons
Consumer	1	15971	6.8	0.003	9948	-
Site	2	3682	1.6	0.18	9949	-
Consumer x Site	2	6343	2.7	0.03	9951	St: south >off
Residual	27	2342				off, north, south: Pl > St

Supplementary Table S16. Fish herbivory, habitat structure and refuge provision.

(a) Indicators of refuge provision (mean ± SD, n = 25) calculated as the canopy height and the rhizome layer corrected by the number of seagrass shoots in the sampling area of 20 cm x 20 cm (=0.04 m^2). Site-specific data on shoot density obtained at the same sites and depth from previous studies, as well as the estimated number of shoots in the sampling area, are also shown. Notes are as follows: (a) Off-vent data on shoot density at 3.3m m depth were obtained from Garrard[7] and corrected by a depth-related variability of 0.8 m according to depth variation found in the same site by Zupo et

al.[8], and (b) Vent data on shoot density at 2.5-3m depth were obtained by averaging values from Donnarumma et al.[9] and Garrard et al.[10]

	Mean shoot density (shoots m-2)	Mean # of shoot in a 0.04 m^2 area	Corrected canopy height (cm shoots)	Corrected rhizome layer (cm shoots)
Off-vent	472[(a)]	19	440 ± 119	141 ± 55
North-vent	648[(b)]	26	496 ± 186	99 ± 51
South-vent	771[(b)]	31	605 ± 177	178 ± 80

(b) Results from one-way PERMANOVA examining differences between sites in structural traits of the seagrass habitat, in fish herbivory as modifier of the seagrass canopy height, as well as in the above-estimated indicators of refuge provision. * (x+0.001)-transformed data.

	Source of variation	df	MS	Pseudo-F	p-value (perm)	Unique perms	Pairwise comparisons
Canopy height	Site	2	128	3.1	0.0498	2812	off > north ~ south
	Residual	72	41				
Rhizome layer	Site	2	82	13	0.0001	7301	off > south > north
	Residual	72	6.3				
Fish herbivory*	Site	2	9981	4.1	0.01	9901	north ~ south > off
	Residual	72	2419				
Corrected canopy height	Site	2	176770	6.6	0.003	9763	south > north ~ off
	Residual	72	26610				
Corrected rhizome layer	Site	2	38834	10	0.0004	9318	south ~ off (p=0.057) > north
	Residual	72	4008				

Supplementary references

1. Kroeker, K. J., Micheli, F., Gambi, M. C. & Martz, T. R. Divergent ecosystem responses within a benthic marine community to ocean acidification. *Proc. Natl. Acad. Sci. USA* **108,** 14515-14520 (2011).
2. Kroeker, K. J., Gambi, M. C. & Micheli, F. Community dynamics and ecosystem simplification in a high-CO_2 ocean. *Proc. Natl. Acad. Sci. USA* **110,** 12721-12726 (2013).
3. Pierrot, D., Lewis, E., Wallace, D. W. R. *MS Excel Program Developed for CO_2 System Calculations. ORNL/CDIAC-105a* (Carbon Dioxide Information Analysis Center, Oak Ridge National Laboratory, U.S. Department of Energy, Oak Ridge, Tennessee, 2006). doi: 10.3334/CDIAC/otg.CO2SYS_XLS_CDIAC105a

4. Dickson, A. G. & Millero, F. J. A comparison of the equilibrium constants for the dissociation of carbonic acid in seawater media. *Deep Sea Res.* **34,** 1733-1743 (1987).
5. Post, D. M. Using stable isotopes to estimate trophic position: Models, methods, and assumptions. *Ecology* **83,** 703-718 (2002).
6. Vizzini, S. et al. Ocean acidification as a driver of community simplification via the collapse of higher-order and rise of lower-order consumers. *Sci. Rep.* **7,** 4018 (2017).
7. Garrard, S. L. The effect of ocean acidification on plant- animal interactions in a *Posidonia oceanica* meadow. PhD these (The Open University Milton Keynes, UK, 2013).
8. Zupo, V., Buia, M.C., Gambi, M. C., Lorenti, M. & Procaccini, G. Temporal variations in the spatial distribution of shoot density in a *Posidonia oceanica* meadow and patterns of genetic diversity. *Mar. Ecol.* **27**, 328-338 (2006).
9. Donnarumma, L., Lombardi, C., Cocito, S. & Gambi, M. C. Settlement pattern of *Posidonia oceanica* epibionts along a gradient of ocean acidification: an approach with mimics. *Mediterr. Mar. Sci.* **15/2**, 498-509 (2014).
10. Garrard, S. et al. Indirect effects may buffer negative responses of seagrass invertebrate communities to ocean acidification. *J. Exp. Mar. Biol. Ecol.* **461**, 31-38 (2014).
